# Endosome-to-TGN Trafficking: Organelle-Vesicle and Organelle-Organelle Interactions

**DOI:** 10.3389/fcell.2020.00163

**Published:** 2020-03-18

**Authors:** Yingfeng Tu, Lin Zhao, Daniel D. Billadeau, Da Jia

**Affiliations:** ^1^Key Laboratory of Birth Defects and Related Diseases of Women and Children, State Key Laboratory of Biotherapy, Department of Paediatrics, West China Second University Hospital, Sichuan University, Chengdu, China; ^2^Division of Oncology Research, Schulze Center for Novel Therapeutics, Mayo Clinic, Rochester, MN, United States

**Keywords:** endosome, TGN, human disease, membrane trafficking, membrane contact site, golgin, WASH complex, TBC1D23

## Abstract

Retrograde transport from endosomes to the *trans*-Golgi network (TGN) diverts proteins and lipids away from lysosomal degradation. It is essential for maintaining cellular homeostasis and signaling. In recent years, significant advancements have been made in understanding this classical pathway, revealing new insights into multiple steps of vesicular trafficking as well as critical roles of ER-endosome contacts for endosomal trafficking. In this review, we summarize up-to-date knowledge about this trafficking pathway, in particular, mechanisms of cargo recognition at endosomes and vesicle tethering at the TGN, and contributions of ER-endosome contacts.

## Introduction

**FIGURE 1:**
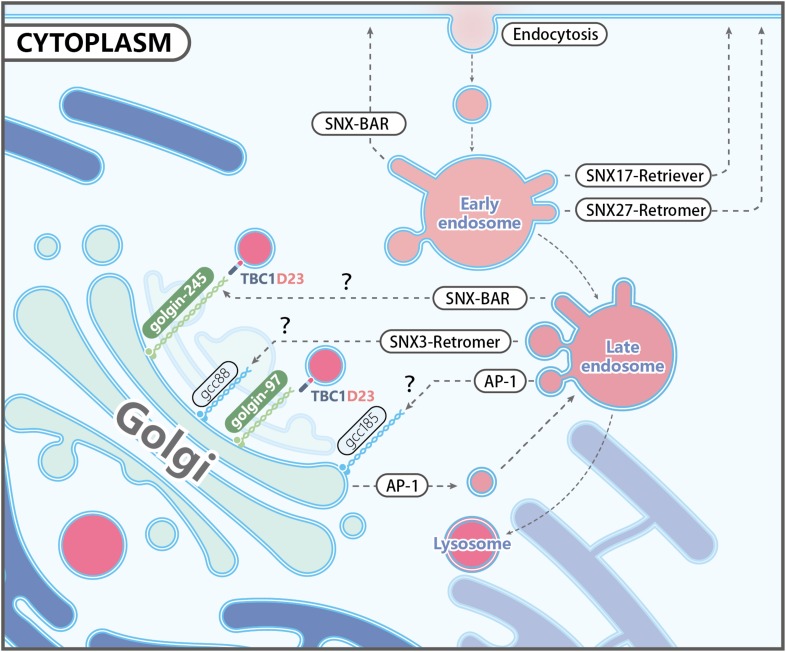
Representative trafficking pathways of transmembrane proteins. Transmembrane receptors are internalized via endocytosis, and some of them are targeted to lysosomes as endosomes mature. Other receptors are recognized by specific coat proteins and sorted to the plasma membrane or the *trans*-Golgi network (TGN), thus evading degradation. Known protein complexes involved in endosome-to-plasma membrane trafficking include SNX17-retriver, SNX27-retromer, and SNX-BARs. Endosome-to-TGN transport depends on cargo recognition mediated by SNX3-retromer, SNX-BARs, or Clathrin/AP1. Other proteins mediating endosome-to-TGN trafficking, including other Adaptor Proteins, Rab9, TIP47, and PACS-1, are not shown for simplicity. The TGN-localized golgin proteins, such as golgin-97, golgin-245, GCC88, and GCC185, are capable of capturing distinct types of endosomal carriers, although the specificity and underlying mechanisms are largely unknown. TBC1D23 acts as an adapter by interacting with golgin-97 and golgin-245, and the WASH complex subunit FAM21 on endosomal vesicles. It should be noted that AP-1 is also found at the TGN, and mediates the bi-directional trafficking between endosomes and the TGN.

Endosomes are central collecting stations in cells where different trafficking pathways converge. Integral membrane proteins, together with their associated proteins and lipids, arrive at endosomes following internalization at the cell surface, and others are transported to endosomes from the *trans*-Golgi network (TGN) (Burd and Cullen, 2014). One of the major functions of endosomes is “sorting,” a term referring to some endosomal proteins and lipids being delivered to the cell surface, via the recycling endosomes, or to the TGN, before reaching the degradative lysosomes (Figure 1). The former pathway is known as endosome-to-plasma membrane recycling, whereas the latter is referred to as endosome-to-TGN retrieval or retrograde transport. Endosomal sorting is crucial for maintaining cellular homeostasis and supporting organism development and growth. Accordingly, many human diseases, including neurological diseases, cancer, and diabetes, have been linked with defects in endosomal functions (Burd and Cullen, 2014; Lucas and Hierro, 2017; McMillan et al., 2017).

The endosome-to-TGN trafficking pathway diverts proteins and lipids away from lysosomal degradation. Mechanistically, this process can be divided into several interconnected steps (Lu and Hong, 2014; Cheung and Pfeffer, 2016; Saimani and Kim, 2017): (1) recognition of endosomal proteins, often in a sequence-dependent manner, by specific protein(s); (2) formation of cargo-enriched endosomal structures/subdomains; (3) endosomal fission leads to the formation of tubulo-vesicular transport carriers; (4) movement of these carriers toward TGN along cytoskeletal tracks; (5) carrier capturing by tethering proteins localized on the TGN; (6) carrier fusion with the Golgi membrane, mediated by the SNARE complex. Recent studies have provided important insights into these steps, including identification of new protein complexes mediating sequence-dependent cargo recognition, identification and characterization of multiple regulatory protein complexes, and discovery of new tethering mechanisms at the TGN (McNally et al., 2017; Shin et al., 2017; Navarro Negredo et al., 2018; Simonetti et al., 2019; Singla et al., 2019).

Endosomes function via contacting and fusing with other endosomes or the incoming vesicles that arrive from the Golgi. In addition, non-fusogenic organelle-organelle interactions likely play a role in endosomal trafficking. Endosomes communicate with other organelles through membrane contact sites (MCSs). Most notably, tubules emanating from the endoplasmic reticulum (ER) contact endosomes within a distance of 30 nm or shorter. The ER-endosome MCSs function to regulate endosome fission, endosome positioning, and cholesterol and Ca^2+^ transfer (Raiborg et al., 2015). In this review, we summarize critical organelle-organelle and organelle-vesicle interactions in the endosome-to-TGN trafficking. We focus on recent advances in understanding recognition of cargo for retrieval, regulation of endosomal fission by ER-endosome contacts, and finally capturing of endosomal carriers at the TGN, in mammalian cells. We will also discuss the relevance of these processes for human development and disease. Due to space limitations, we are unable to cover every aspect of this trafficking pathway. Readers are invited to look through several recently published reviews that cover aspects not discussed here (Burd and Cullen, 2014; Lu and Hong, 2014; Raiborg et al., 2015; Liu, 2016; Lucas and Hierro, 2017; McMillan et al., 2017; Cullen and Steinberg, 2018).

## Cargo Recognition at Endosomes

The endosome-to-TGN trafficking pathway is one of two major routes that diverge from lysosomal degradation. Endosomal proteins are delivered to the TGN through two distinct itineraries: from either the early endosome/recycling endosome or the late endosome (Ghosh et al., 1998; Mallet and Maxfield, 1999). Proteins transiting this pathway include members of the Vps10 domain family cargo receptors (such as sortilin and SorLA/SorL1) (Nielsen et al., 2001; Willnow and Andersen, 2013), cation-dependent or cation-independent mannose-6-phosphate receptor (CD-MPR or CI-MPR) (Meyer et al., 2000; Schweizer et al., 2000; McKenzie et al., 2012), enzymes such as endoprotease furin and carboxypeptidase D (Varlamov and Fricker, 1998), and TGN38/46 (Ghosh et al., 1998). Furthermore, bacterial and plant toxins, such as Shiga toxin, Cholera toxin, and Ricin, hijack this pathway for their intracellular traveling before reaching the ER (Sandvig and van Deurs, 2002; Mukhopadhyay and Linstedt, 2012).

A number of proteins have been shown to mediate sequence-specific sorting to the TGN, including retromer (Seaman, 2018), SNX-BARs (Kvainickas et al., 2017a; Simonetti et al., 2017, 2019), clathrin and adaptors (Hirst et al., 2012, 2013), Rab9 GTPase and TIP45 (Carroll et al., 2001), and PACS-1 (Wan et al., 1998).

### Retromer

Retromer was initially found to mediate endosome-to-TGN transport of Vps10 in yeast (Seaman et al., 1998; Nothwehr et al., 2000). Similarly, mammalian retromer (VPS35/VPS26/VPS29) has been shown by multiple groups to mediate the transport of CI-MPR, the functional equivalent of Vps10, and other cargo proteins, likely through a direct interaction (Arighi et al., 2004; Seaman, 2004). It is reported that a sequence comprising Trp-Leu-Met (WLM) present in the cytoplasmic tail of CI-MPR is required to associate with retromer and for the endosome-to-Golgi retrieval of CI-MPR (Seaman, 2007).

Multiple distinct cargo-recognizing mechanisms have been discovered. For instance, yeast Vps10 contains a bipartite signal that is recognized by the retromer subunit Vps35 and Vps26 (Suzuki et al., 2019). In mammals, the SNX3-retromer complex mediates the transport of wntless, DMT1 (divalent metal transporter 1), and CI-MPR, via recognizing the aromatic, hydrophobic motif in their cytoplasmic tails (Harterink et al., 2011; Zhang et al., 2011; Harrison et al., 2014; Lucas et al., 2016; Cui et al., 2019). Crystal structure of the quaternary VPS35-VPS26-SNX3-DMT1 tail complex revealed that VPS26 and SNX3 cooperate to mediate cargo recognition (Lucas et al., 2016). Cargo recognition is achieved through a different mechanism for another cargo protein, SorLA. It has been shown that VPS26 on its own binds to the FANSHY sorting motif presented in SorLA, although structural evidence remains to be established (Fjorback et al., 2012).

### SNX-BAR Proteins

The SNX-BAR proteins belong to the PX (phox-homology) domain and SNX (sorting nexin) protein family (Teasdale and Collins, 2012). They contain a BAR (Bin/Amphiphysin/Rvs) domain, in addition to the PX domain. The retromer-related SNX-BAR proteins (referred to as SNX-BARs herein) function as a heterodimer, through the interaction between SNX1 or SNX2, and SNX5 or SNX6 or SNX32 (Carlton et al., 2004; Wassmer et al., 2007). Traditionally, SNX-BARs are regarded as a membrane-binding module to facilitate retromer-mediated cargo recognition and retrieval. The presence of two membrane-interacting domains in SNX–BARs enables coincidence detection of PI3P (phosphatidylinositol 3-phosphate), by the PX domain of SNX1/2, and membrane curvature, by the BAR domain (Teasdale and Collins, 2012). Recently, two independent studies reveled that SNX-BARs can directly associate with CI-MPR to mediate its retrieval (Kvainickas et al., 2017a; Simonetti et al., 2017). Moreover, these studies also showed that deletion of VPS35, unlike deletion of SNX-BARs, did not cause a pronounced defect in CI-MPR trafficking (Kvainickas et al., 2017a; Simonetti et al., 2017). Cargo proteins recognized and transported by SNX-BARs are not limited to CI-MPR, and now the list of cargoes includes IGF1R, SEMA4C, and dozens of other integral membrane proteins, which undergo either endosome-to-TGN or endosome-to-plasma membrane trafficking (Kvainickas et al., 2017a; Simonetti et al., 2017, 2019; Bareja et al., 2018).

The SNX-BAR proteins recognize a bipartite sorting signal [ΦXΩXΦ(X)nΦ, Φ, hydrophobic residues; X, any residue; Ω, aromatic residues] in cargo proteins (Simonetti et al., 2019). Interestingly, the WLM motif in the cytoplasmic tail of CI-MPR is part of the soring signal recognized by SNX-BARs. The PX domain of SNX5/SNX6/SNX32 contains a long insertion relative to other PX domains, and does not bind to phosphoinositides, such as PI3P (Chandra et al., 2019). Instead, this insertion, together with a proximal β-strand, constitute the binding site for the sorting signal, which forms two antiparallel β-strands (Simonetti et al., 2019). Interestingly, structure of the sorting signal in the complex resembles that of the Chlamydia trachomatis effector protein IncE (Elwell et al., 2017; Paul et al., 2017; Sun et al., 2017).

Is CI-MPR a cargo of retromer, or SNX-BARs? Current evidence suggests two possible models. The first model suggests that retromer and SNX-BARs mediate two independent pathways for CI-MPR retrieval. Supporting this model is the observation that retromer and SNX3, and SNX-BARs, mediate CI-MPR transport in carriers that depend on different tether proteins on the TGN (see later) (Cui et al., 2019). The second model supports that SNX-BARs play a dominant role in CI-MPR trafficking, whereas retromer regulates CI-MPR trafficking through interacting with TBC1D5 and modulating the activity of Rab7 GTPase (Jia et al., 2016; Jimenez-Orgaz et al., 2018; Kvainickas et al., 2019). Consistent with this model, Steinberg et al. (2013) showed that deletion of VPS35, although not having a pronounced effect on the subcellular localization of CI-MPR, altered the transportation kinetics of endocytosed CI-MPR to the TGN (Kvainickas et al., 2017a). Distinguishing these two models represent an exciting direction in the field.

### Clathrin and Adapters

In addition to retromer and SNX-BARs, clathrin and its adaptor proteins are also involved in endosome-to-TGN trafficking. Clathrin associates with two types of adaptor proteins: tetrameric and monomeric. The former includes five Adaptor Proteins: AP-1, AP-2, AP-3, AP-4, and AP-5. All of them, excepted for AP-2, participates in endosomal trafficking (Park and Guo, 2014). The monomeric adaptor proteins include epsinR and GGA (Golgi-localized, γ-adaptin ear-containing, Arf-binding). Among all the adaptor proteins associating with clathrin, AP-1 and epsinR have the most established roles in CI-MPR retrieval to the TGN. Unlike retromer, AP-1 and epsinR localize on both endosomes and the TGN (Meyer et al., 2000). Depletion of AP-1 or epsinR results in a dispersed MPR localization pattern, consisting with defects in endosome-to-TGN transport (Meyer et al., 2000; Hirst et al., 2004; Saint-Pol et al., 2004; Robinson et al., 2010). These results are further supported by rapid depletion of AP-1 by the “knocksideways” system, which reveals AP-1 affects a large number of proteins, including lysosomal hydrolases and their receptors (MPR), various SNAREs, and many integral membrane proteins (Hirst et al., 2012). AP1 could directly bind cargos by interacting with YXXΦ and [DE]XXXL[LI] motifs in their cytosolic tails (Rapoport et al., 1998). In addition to AP-1, AP-5 is also implicated in the retrieval of CI-MPR (Hirst et al., 2013). Although retromer, SNX-BARs, and clathrin are all involved in the endosome-to-TGN trafficking, it remains obscure whether they function together or separately, and whether they function sequentially or concurrently.

## Er-Endosome Contact Sites and Endosome Fission

### ER-Endosome Contact Sites

The ER is a continuous network of tubules and cisternae that have versatile cellular functions, including protein synthesis and transport, lipid metabolism, calcium storage, and stress response. This network spreads throughout the cytoplasm, physically separated from other membrane compartments but functionally connected with organelles of the endocytic pathway, the nuclear membrane, and the plasma membrane (Wu et al., 2018). In addition to vesicular trafficking, MCSs have emerged as an alternative means of inter-organelle communication between the ER and other intracellular membranes. One developing theme is that each type of MCS has a unique molecular composition with unique functions (Raiborg et al., 2015; Wu et al., 2018).

### ER-Endosome Contact Sites Regulate Endosome Fission

Endosome fission is the step whereby membrane carriers, including vesicles and tubules, bud and split from endosomes. It is critical for recycling of cargoes to the plasma membrane or TGN, and important for endosome maturation. ER tubules were initially found at the sites of mitochondria division (Friedman et al., 2011), and Rowland et al. demonstrated that ER-endosome contact sites are also required for endosome fission (Rowland et al., 2014). ER tubules make contact with the endosome, marked by the WASH complex subunit FAM21, just before the fission event (Rowland et al., 2014). Consistently, altered shape and dynamics of ER by overexpression of Reticulon 4a, which is known to generate ER tubules, results in a significantly decreased number of endosome buds undergoing fission (Shibata et al., 2008; Rowland et al., 2014).

**FIGURE 2:**
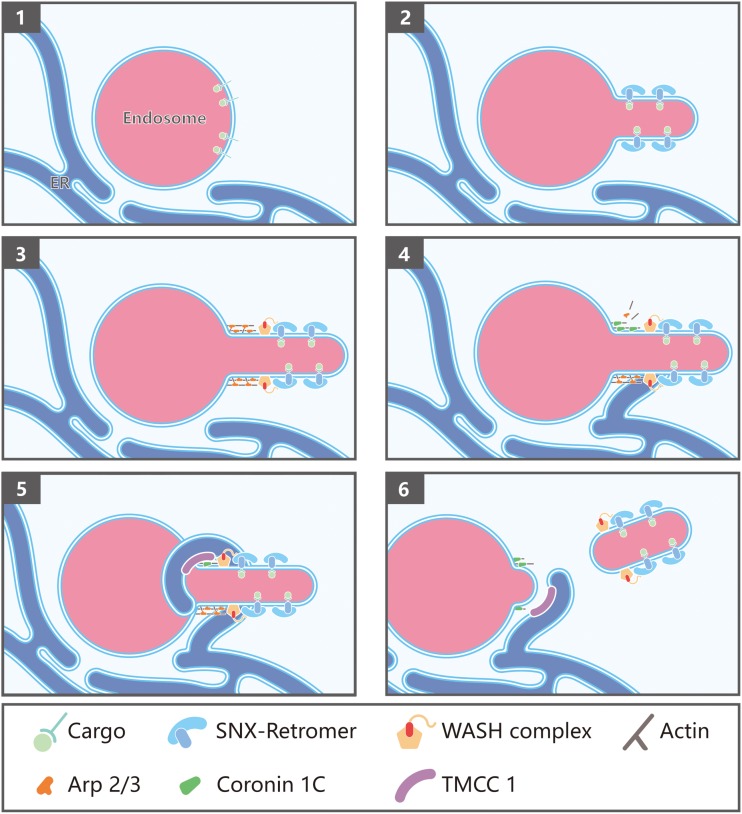
Endoplasmic reticulum (ER)-endosome membrane contact sites in endosome fission: (1) Endosomes contain internalized transmembrane proteins or proteins delivered from the Golgi; (2) protein complexes, such as SNXs and retromer, recognize and concentrate specific cargo, and promote membrane remodeling; (3) retromer recruits the WASH complex via a direct interaction with the FAM21 subunit, which in turn promotes the assembly of branched actin via Arp2/3; (4) Coronin 1C associates with Arp2/3-mediated actin patches present on endosome buds; (5) Coronin 1C interacts with the ER membrane protein TMCC1 in order to recruit the ER to the Coronin 1C-labeled endosomal domains; (6) endosome fission occurs, likely via fission factors whose identity remains to be determined. Possible candidates include Dynamin2 and the EHD family proteins.

Given the immediate establishment of ER-endosome contact sites prior to fission, these contact sites might be extremely transient, thus hampering the identification of its molecular composition. Using a proximity biotinylation assay in which FAM21 was tagged, Hoyer et al. identified the ER transmembrane protein TMCC1 as a critical protein mediating the ER-endosome contact (Hoyer et al., 2018). Depletion of TMCC1 specifically impairs fission of endosomal tubules and subsequent cargo sorting, but does not alter the formation of these tubules, emphasizing the importance of ER-endosome MCSs in endosome fission. Hoyer et al. further demonstrated that endosome-localized Coronin 1C is involved in ER-mediated endosome fission (Hoyer et al., 2018). Coronin 1C is an actin-binding protein that has been shown to accumulate at sites of Arp2/3-generated actin patches on endosomes (Puthenveedu et al., 2010). Interestingly, depletion of Coronin 1C prevented TMCC1 recruitment leading to a dramatic reduction in ER-endosome contacts and defective endosome fission of WASH-labeled endosomal buds (Hoyer et al., 2018; Figure 2). Since Coronin proteins are known to mediate F-actin disassembly by displacing Arp2/3 complexes, Coronin 1C might function to not only recruit TMCC1/ER MCSs, but to also simultaneously disassemble branched F-actin at the site of endosomal fission (Figure 2).

In addition to TMCC1 and Coronin 1C, the ER-endosome MCSs can be also mediated by ER protein VAP (vesicle-associated membrane protein-associated protein, including VAP-A and VAP-B) and two proteins localized on endosomes: SNX2 and OSBP (Oxysterol Binding Protein) (Dong et al., 2016). Loss of VAP or OSBP results in increased PI4P and actin levels on endosomes, and leads to defective endosome-to-Golgi traffic (Dong et al., 2016). It remains to be determined whether the loss of VAP or OSBP decreases endosome budding or subsequent bud fission.

From a mechanistic standpoint, it is unclear how ER-endosome MCSs contribute to scission of endosomal tubules. It is likely that some fission proteins are recruited to these sites to facilitate scission. Interesting candidates include Dynamin2 and the EHD (Eps15 homology domain) proteins, which could provide the mechanochemical force required for the fission. Dynamin2 has been reported to interact with the WASH complex, indicating a putative role for the scission of tubules (Derivery et al., 2009). The human genome encodes four EHD proteins, which share the conserved EHD domain. Structural studies of EHD2 reveals that the EHD domain forms ring-like oligomers around tubules, similar to the Dynamin GTPase (Daumke et al., 2007). It has been shown that EHD1 associates with retromer, and stabilizes the membrane tubules generated by SNX1 (Gokool et al., 2007; Zhang et al., 2012). Thus, Dynamin2 and EHD proteins may function together with the WASH complex to promote endosomal fission at the ER-endosome MCSs.

### ER-Endosome MCSs and Human Diseases

Emphasizing the importance of ER-endosome MCSs for human health is the observation that mutations or aberrant expression of corresponding genes have been linked with many types of human diseases. Hereditary spastic paraplegia (HSP) is a group of inherited diseases characterized by progressive stiffness in the lower limbs. Among the over 60 genes that have been linked with HSP (Hensiek et al., 2015), many of them are implicated in the formation of ER-endosome MCSs, such as REEP1, WASH complex subunit strumpellin, ER protein protrudin, and spastin (Beetz et al., 2006, 2008; Mannan et al., 2006; Valdmanis et al., 2007). For instance, spastin is an AAA family ATPase and functions as a microtubule severing protein (Roll-Mecak and Vale, 2008). The ER-localized spastin isoform mediates ER-endosome MCSs through interacting with the ESCRT-III protein IST1, which is critical for sorting of mannose 6-phosphate receptor and consequently lysosomal enzyme trafficking (Allison et al., 2017). Accordingly, abnormal lysosomal morphology has been observed in neurons and other types of cells that are depleted of not only spastin, but also REEP1 and strumpellin, suggesting that aberrant ER-endosome contacts likely contribute to the pathogenesis of HSP (Allison et al., 2017).

In addition to HSP, dysregulation of ER-endosome MCSs are implicated in other diseases. For instance, Niemann–Pick diseases are a group of metabolic diseases, in which large quantities of lipids accumulate in the spleen, liver, brain, and other parts of the body. Mutations of NPC1 (Niemann-Pick C1), which mediates the ER-late endosome contacts, and NPC2 (Niemann-Pick C2), result in failed cholesterol transport from the endosomal lumen and lead to Niemann–Pick disease (Carstea et al., 1997; Goldstein and Brown, 2001; Sleat et al., 2004).

## Capturing Endosomal Carriers at the TGN

Transported vesicles destined for the Golgi are captured by tethering factors at a long distance (could extend for 100–400 nm), before subsequent vesicle docking and fusion mediated by the SNARE complex. Tethering factors localized on the Golgi can be divided into two classes: large oligomeric complexes including Golgi-associated retrograde protein (GARP) and conserved oligomeric Golgi (COG), and homodimeric golgin proteins (Whyte and Munro, 2002; Yu and Hughson, 2010).

### GARP and COG

Both GARP and COG are recruited to the Golgi via interaction with small GTPases of the Rab and Arl families. COG is an octamer localized in all Golgi cisternae and plays a role in the retrieval of resident proteins between stacks (Zolov and Lupashin, 2005; Ungar et al., 2006). COG mutations have been linked with congenital disorders of glycosylation (CDG) (Zeevaert et al., 2008). The GARP complex (VPS51, VPS52, VPS53, and VPS54), mainly found at the TGN, is involved in transport of cargoes including the late-Golgi SNAREs and CI-MPR (Conibear et al., 2003; Perez-Victoria et al., 2008). This trafficking pathway is further stimulated by amino acids, requiring the small GTPase Arl5 and the Ragulator complex. Arl5 enhances the membrane recruitment of the GARP complex, and Ragulator may function as a guanine nucleotide exchange factor for Arl5 (Shi et al., 2018).

### Golgins

Golgins represent another class of tethering proteins at the Golgi. Highly conserved among eukaryotes, golgins feature a long coiled-coil structure. The number of golgin-encoding genes has expanded during evolution, with 5 and more than 10 golgin-encoded genes in the *S. cerevisiae* and human genomes, respectively (Munro, 2011). The large number of golgin proteins is consistent with their diverse cellular functions. Using a mitochondrial re-location assay, Munro et al., found that golgin proteins could capture distinct sets of Golgi-bound transport vesicles (Wong and Munro, 2014). For instance, GM130 and GMAP-210 that localize in the *cis*-Golgi are capable of tethering vesicles arriving from the ER. The TGN-localized golgins, golgin-97, golgin-245, and GCC88, capture vesicles from endosomes; GCC185 did not capture vesicles in the assay (Wong and Munro, 2014). However, a separate study showed that GCC185 contains an AP-1 binding site and likely involves tethering of AP-1-decorated vesicles (Brown et al., 2011).

Different golgin proteins localize in different parts of the Golgi stack, and four mammalian golgins localize at the TGN: golgin-97, golgin-245, GCC88 and GCC185 (Figure 1). They share a conserved domain of about 80 residues at their carboxyl terminus, called the GRIP domain. The GRIP domain binds to the activated Arl1 GTPase localized at the TGN, which is critical for the Golgi-targeting of these golgins (Witkos and Lowe, 2015; Gillingham and Munro, 2016). In addition to Arl1 GTPase, these golgins also harbor binding sites for a variety of proteins, including AP-1, Rab GTPases, motor proteins and SNAREs (Derby et al., 2007; Burguete et al., 2008; Miller et al., 2009; Brown et al., 2011). These interactions allow golgins to capture specific transport vesicles from a long distance, as some golgins have a length of over 100 nm, and subsequently tether vesicles to the destination membrane.

One emerging concept in the field is that there are some degrees of crosstalk between the proteins involved in cargo recognition and carrier formation, and those involved in vesicle tethering (Figure 1). For instance, Cui et al. showed that SNX3- and retromer-dependent CI-MPR carriers are recognized and captured by GCC88, but not by golgin-97 or golgin-245 (Cui et al., 2019). On the other hand, SNX-BARs mediate CI-MPR trafficking in carriers that are captured by golgin-245 (Cui et al., 2019). It will be highly informative to determine how different types of carriers are recognized by different tethers.

### TBC1D23

Three golgins mediate endosome-to-TGN transport: golgin-97, golgin-245, and GCC88 in the mitochondrial re-location assay (Wong and Munro, 2014). How these golgin proteins capture transport vesicles from endosomes remained unclear until recently. Earlier studies suggested that the N-termini of golgins are required to capture correct vesicles by interacting with vesicle-specific proteins or lipids (Wong and Munro, 2014). Using the mitochondrial re-location and proximity biotinylation assays, Shin et al. identified that the N-termini of golgin-97 and golgin-245, but not GCC88, interact with TBC1D23 (Shin et al., 2017). TBC1D23 also interacts with the WASH complex subunit FAM21, which is localized on vesicles derived from endosomes (Shin et al., 2017). Thus, TBC1D23 functions as an adapter to bridge endosomal vesicles with the TGN.

**FIGURE 3:**
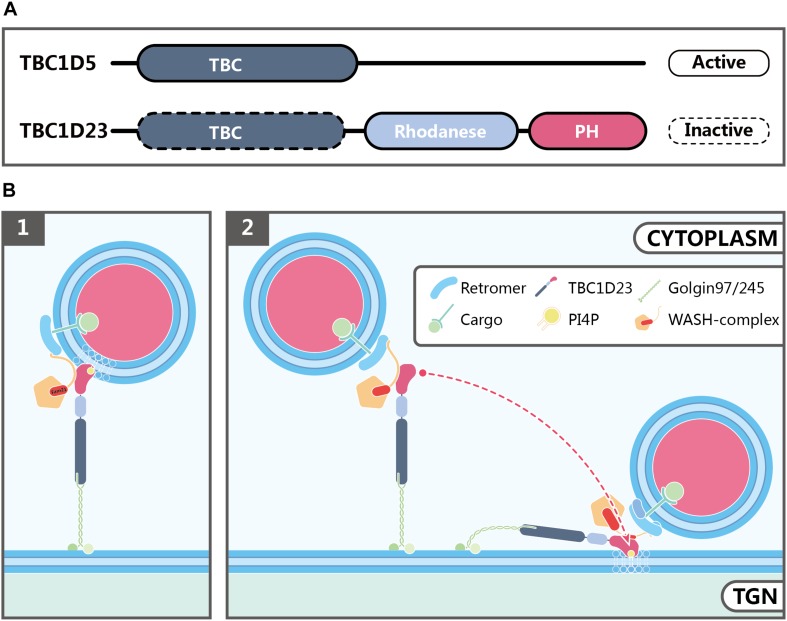
Possible functions of TBC1D23 in endosome-to-TGN trafficking. **(A)** Domain structures of TBC1D5 and TBC1D23. Although both proteins possess a TBC domain, TBC1D5, but not TBC1D23, is an active GAP for Rab GTPases. In addition to the TBC domain, TBC1D23 also contains a rhodanese-like domain, and a C-terminal domain, which is structurally similar to phospholipid-binding PH domains despite bearing little sequence similarity. **(B)** Models showing how TBC1D23 regulates endosome-to-TGN trafficking by interacting with FAM21 and specific phosphoinositides, such as PI4P. FAM21 associates with WASH, TBC1D23 and retromer via its N-terminus, the middle region, and C-terminus, respectively. Whereas the C-terminal domain of TBC1D23 engages with PI4P, in addition to FAM21, future studies will be needed to address whether such interactions help to capture the PI4P-positive endosomal vesicles by TBC1D23 (1), or promote the tethering of TBC1D23-bound vesicles to the TGN (2).

TBC1D23 is a member of the Tre2-Bub2-Cdc16 (TBC) family and can be found in most eukaryotic organisms, except for fungi and plants (Shin et al., 2017). The wide distribution and ubiquitous expression of TBC1D23 in various tissues and cells are consistent with its fundamental roles in biology and in development. Two human TBC1D23 isoforms are described so far, which differ by one exon (exon 15). As a result, the longer isoform including this exon encodes a protein with 699 residues, and the shorter one encodes 684 residues (Marin-Valencia et al., 2017). TBC1D23 contains three functional domains: an N-terminal TBC domain, a Rhodanese-like domain in the middle, and an C-terminal domain which is shown to be structurally similar to a Pleckstrin homology (PH) domain (Figure 3A; Wang et al., 2018; Huang et al., 2019). Its TBC domain interacts with the N-terminal 21 residues from golgin-97 or golgin-245 (Shin et al., 2017). Members of the TBC family often function as Rab GTPase-activating proteins (GAPs), and contain two conserved catalytic residues, Arg and Gln within their GAP domain (Pan et al., 2006). For instance, TBC1D5 is a GAP for both Rab7a and Rab7b, and functions to regulate retromer-dependent trafficking, and to regulate late endosomal and lysosomal functions (Figure 3A; Mukhopadhyay et al., 2007; Seaman et al., 2009; Jia et al., 2016; Borg Distefano et al., 2018; Jimenez-Orgaz et al., 2018; Kvainickas et al., 2019). In contrast, TBC1D23 lacks both essential catalytic residues, and is catalytically inactive (Marin-Valencia et al., 2017). Proteins with a rhodanese domain can possess sulphurtransferase (such as TSTD1) or phosphatase (such as CDC25) activity (Bordo and Bork, 2002). It remains to be determined whether the rhodanese domain of TBC1D23 possesses any enzymatic activities; if it does, how the enzymatic activities contribute to its functions in membrane trafficking remains to be determined. Finally, we recently solved the crystal structure of the TBC1D23 C-terminal domain, and showed that it selectively binds to phosphoinositides, including PI4P, and the FAM21 subunit of the WASH complex, using opposite surfaces (Huang et al., 2019). Whereas these observations are interesting, additional work is needed to address whether such interactions help to capture the PI4P-positive endosomal vesicles by TBC1D23, or promote the tethering of TBC1D23 and bound vesicles by the TGN (Figure 3B).

Emphasizing the importance of TBC1D23 in human development, homozygous mutations of the TBC1D23 gene has recently been found in patients diagnosed with pontocerebellar hypoplasia (PCH) (Ivanova et al., 2017; Marin-Valencia et al., 2017; Harripaul et al., 2018). These mutations result in truncated proteins that are missing the C-terminal domain. PCH is a group of neurological disorders, characterized by the impaired development of the brain, especially the pons and the cerebellum. Other features of PCH patients include delayed development, microcephaly, movement problems, and intellectual disability. Interestingly, most of the genes linked with PCH are involved in RNA processing (Namavar et al., 2011; Rudnik-Schoneborn et al., 2014), unlike TBC1D23. Using cellular assays and zebrafish models, we demonstrated a strong correlation between cellular and zebrafish phenotypes caused by different TBC1D23 mutants, indicating that mis-regulation of cargo trafficking from endosomes, at least partially, contributes to the development of PCH (Huang et al., 2019). Future studies will be necessary to determine which cargo proteins are most severely affected by TBC1D23 deletion or mutations, which could provide fresh insights into the pathogenesis of certain types of PCH.

### WDR11

In addition to goglin-97, golgin-245, and FAM21, TBC1D23 also interacts with a trimeric complex, consisting of WDR11, FAM91A, and C17orf75, through a region between the rhodanese and PH domains (Borner et al., 2014; Shin et al., 2017; Navarro Negredo et al., 2018). The WDR11 complex is proposed to function together with TBC1D23 and the clathrin adaptor, AP-1, to promote endosome-to-TGN trafficking (Navarro Negredo et al., 2018). One possibility is that WDR11 localizes on the TGN and functions to capture vesicles containing AP-1-dependent cargo; the other possibility is that WDR11 associates with the vesicle, and its interaction with TBC1D23 promotes vesicle tethering at the TGN (Navarro Negredo et al., 2018). Either scenario is consistent with some tethering roles of TBC1D23 and the WDR11 complex, although the exact mechanisms remain to be determined.

Intriguingly, mutations of WDR11 have been found in patients with congenital hypogonadotropic hypogonadism (CHH) and Kallmann syndrome (KS), developmental disorders characterized by delayed puberty and infertility (Kim et al., 2010). WDR11 is essential for normal ciliogenesis, by being involved in the Hedgehog (Hh) signaling pathway (Kim et al., 2018). However, it is unclear whether TBC1D23 is also involved in these processes.

## Wash Complex

One molecule recurring through many distinct processes of endosomal trafficking is the evolutionarily conserved WASH complex, which promotes endosomal branched actin polymerization (Derivery et al., 2009; Gomez and Billadeau, 2009; Jia et al., 2010). In cells, WASH forms a stable pentameric complex together with four other proteins, including FAM21 (WASHC2), CCDC53 (WASHC3), SWIP (Strumpellin and WASH1-interacting protein or WASHC4), and Strumpellin (WASHC5) (Derivery et al., 2009; Jia et al., 2010). The WASH complex predominantly localizes to endosomes and functions to regulate multiple endosomal trafficking pathways. Among them, the best-characterized function of WASH is to regulate retromer-dependent trafficking through an association with retromer, via a direct interaction between the retromer subunit VPS35 and FAM21 (Harbour et al., 2010; Jia et al., 2012). WASH modulates endosome-to-TGN or endosome-to-plasma membrane transport of many endosomal proteins, such as CI-MPR, the β2-adrenoceptor (β2AR), the glucose transporter GLUT1, and transferrin receptor (TfnR) (Derivery et al., 2009; Puthenveedu et al., 2010; Zech et al., 2011; Gomez et al., 2012; Piotrowski et al., 2013; Phillips-Krawczak et al., 2015). Recent studies demonstrate that the WASH complex also functions to orchestrate retromer-independent trafficking, by cooperating with retriever and the CCC complex (McNally et al., 2017). In addition to retromer, HRS (hepatocyte growth factor–regulated tyrosine kinase substrate) also plays a role in regulating endosomal localization of WASH, although it is unclear whether the interaction between WASH and HRS is direct (MacDonald et al., 2018). It is reported that WASH and HRS cooperate to regulate endosomal recycling of epidermal growth factor receptor and the matrix metalloproteinase MT1–MMP (MacDonald et al., 2018). The WASH complex also associates with BLOC-1 (biogenesis of lysosomal organelles complex-1), and may play a role in the formation of melanosomes (Monfregola et al., 2010; Ryder et al., 2013). In all of the above processes, WASH likely functions to promote actin polymerization and to provide the force required for the formation of endosomal recycling or retrieval subdomains, or for vesicle budding and scission. The WASH complex, especially FAM21, also likely serves as a recruiting hub for additional proteins involved in the trafficking of proteins to specific subcellular destinations (Seaman et al., 2013; Wang et al., 2018).

The WASH complex possesses additional functions. It has been shown that WASH defines endosome fission sites by locally activating the Arp2/3 complex to generate branched F-actin, and the subsequent recruitment of Coronin 1C, which facilitates interaction with TMCC1 leading to ER-endosome contact (Rowland et al., 2014; Hoyer et al., 2018). Finally, the interaction between endosomal-localized FAM21 and Golgi-localized TBC1D23 promotes vesicle tethering at the TGN (Shin et al., 2017; Huang et al., 2019). How can one protein complex possess so many diverse functions? Part of the answer lies in the fact that FAM21 is a large protein with over 1300 residues. Although slightly over 200 residues within the N-terminus of FAM21 are sufficient for assembly of the WASH pentameric complex, the C-terminal tail of FAM21 harbors 21 repeats of the LFa motif (L-F-[D/E]3-10-L-F), and is capable of engaging several proteins, including retromer, TBC1D23, CCC complex, CapZ, and FKBP15 among others (Hernandez-Valladares et al., 2010; Jia et al., 2010, 2012; Takeda et al., 2010; Harbour et al., 2011, 2012; Freeman et al., 2014; Phillips-Krawczak et al., 2015; Kvainickas et al., 2017b; Shin et al., 2017). Interestingly, although both retromer and TBC1D23 bind to LFa motifs within the FAM21 tail, their different sequence preference allows them to bind non-overlapping motifs (Huang et al., 2019).

Deletion of WASH in mice leads to embryonic lethality, emphasizing the indispensable role of the WASH complex in mammalian development (Gomez et al., 2012; Xia et al., 2013). Interestingly, WASH deletion in *Drosophila* does not result in lethality, likely due to a compensatory effect of related genes (Verboon et al., 2018). Furthermore, mutations in Strumpellin and SWIP have been linked with multiple neurological disorders, including intellectual disability, hereditary spastic paraplegia, and Ritscher-Schinzel/3C syndrome (Valdmanis et al., 2007; Ropers et al., 2011; Vardarajan et al., 2012; de Bot et al., 2013; Elliott et al., 2013). Finally, an early onset Parkinson’s Disease (PD) mutation in VPS35 (D620N) was shown to compromise the interaction between the WASH complex and retromer, and to impair endosomal trafficking as well as autophagy (Vilarino-Guell et al., 2011; Zimprich et al., 2011; Follett et al., 2014; McGough et al., 2014; Miura et al., 2014; Zavodszky et al., 2014). Altogether, these studies highlight that regulation of endosomal trafficking via fine-tuning actin polymerization is essential for mammalian development, especially for neuronal development.

## An Evolutionary Perspective

Although the endosome-to-TGN trafficking is absolutely conserved in eukaryotes, it is interesting to note the divergence of many important genes (Table 1). Whereas retromer, SNX3, SNX-BARs are conserved in the eukaryotic kingdom, many genes encoding proteins with regulatory functions, such as the WASH complex and EHD1, are missing in *S. cerevisiae* (Seaman, 2012). Similarly, whereas mammals have four golgin proteins localized at the TGN, yeast only has one, IMH1 (Munro, 2011). IMH1 is known to participate Ypt6-mediated, the yeast homolog of mammalian Rab6 GTPase, endosome-to-TGN transport (Chen et al., 2019). However, yeast does not have clear homologs of TBC1D23 or WDR11, suggesting that IMH1 exert its functions through a mechanism distinct from that of golgin-97 and golgin-245.

**TABLE 1:** Conservation of selected genes involved in endosome-to-TGN trafficking.

		*Homo sapiens*	*Saccharomyces cerevisiae*
Coats and related proteins	Retromer	√	√
	TBC1D5	√	
	SNX-BAR	√ (SNX1/SNX2 + SNX5/SNX6/SNX32)	√ (Vps5 + Vps17)
	SNX3	√	√
	AP-1	√	√
Tethers and related proteins	Golgin-97	√	
	Golgin-245	√	Imh1
	GCC88	√	
	GCC185	√	
	TBC1D23	√	
	WDR11	√	
Other	WASH complex	√	
	EHD1	√	

Furthermore, although retromer and SNX-BARs are conserved between yeast and mammals, they show marked differences in many aspects. First, yeast retromer is a stable pentameric complex, consisting of the Vps35-Vps26-Vps29 trimer and the SNX-BAR proteins Vps5 and Vps10 (Seaman et al., 1998). However, in mammals and other higher metazoans, the VPS35-VPS26-VPS29 trimer [referred to as retromer in metazoans (Burd and Cullen, 2014)] and the SNX-BAR dimer function as independent entities, and are only loosely connected with each other. Second, as separate entities, both mammalian VPS35-VPS26-VPS29 trimer and the SNX-BAR dimer have gained additional functions. For instance, mammalian VPS35-VPS26-VPS29 trimer associates with SNX3 or SNX27 to form functionally distinct entities, independent of SNX-BARs (Harterink et al., 2011; Temkin et al., 2011; Steinberg et al., 2013; Gallon et al., 2014). Similarly, mammalian SNX-BAR dimer, but not the yeast Vps5 and Vps10 dimer, are directly involved in sequence-dependent cargo recognition (Kvainickas et al., 2017a; Simonetti et al., 2017, 2019). Lastly, although yeast retromer is a known regulator of endosome-to-Golgi trafficking, both mammalian retromer and SNX-BARs play critical roles in both endosome-to-TGN and endosome-to-plasma membrane recycling. Thus, although yeast represents an excellent model to investigate the endosome-to-TGN trafficking, cautions must be taken when knowledge learned from different organisms are considered together.

## Conclusion

The past a few years have witnessed great advances in the field of endosomal biology. First, multiple types of ER-endosome MCSs have been characterized, with each possessing unique molecular compositions and functions (Raiborg et al., 2015; Wu et al., 2018). ER-endosome contact sites are known to regulate many aspects of endosomal functions, ranging from endosome positioning and fission, to lipid and ion exchange. Second, several new protein complexes, including the SNX17-retriever complex and SNX-BARs, have been discovered to mediate sequence-dependent cargo retrieval and recycling (Kvainickas et al., 2017a; McNally et al., 2017; Simonetti et al., 2017, 2019). Third, the mechanisms by which endosomal vesicles are captured by the Golgi are emerging (Cheung et al., 2015; Shin et al., 2017; Navarro Negredo et al., 2018). These advances have greatly expanded our understanding of endosomal trafficking, and provided insight into mechanisms contributing to human disease.

The fast progress made in this field suggests that we can anticipate more exciting developments in the new decade. However, despite the huge leap forward in the field, we are still left with many important questions. For those studying ER-endosome contact, a big question is whether other types of contact sites exist, and what are their functions? How are the dynamics of ER-endosome contact sites regulated? How do ER-endosome MCSs promote endosome fission from a mechanistic standpoint? For those interested in vesicular trafficking, it remains to be determined the precise roles and relationship of different molecules involved in cargo recognition and carrier formation, such as retromer, SNX-BARs, and clathrin/AP-1. For most cases, it is still unclear how tethering proteins recognize specific vesicles. Whereas long tethering proteins can capture vesicles as far away as a few hundred nanometers, the SNARE complex mediates vesicle fusion at a much shorter distance (<10 nm). How does the vesicle tethering step connect with the fusion step? Lastly, as our knowledge increases regarding the molecular mechanisms by which dysregulation of endosomal functions contribute to human disease, the translation of these basic discoveries into the clinic are likely to be rapidly approaching.

## Author Contributions

All authors listed have made a substantial, direct and intellectual contribution to the work, and approved it for publication.

## Conflict of Interest

The authors declare that the research was conducted in the absence of any commercial or financial relationships that could be construed as a potential conflict of interest.
